# Arfaptin-1 Negatively Regulates Arl1-Mediated Retrograde Transport

**DOI:** 10.1371/journal.pone.0118743

**Published:** 2015-03-19

**Authors:** Lien-Hung Huang, Wei-Chung Lee, Shu-Ting You, Chia-Chen Cheng, Chia-Jung Yu

**Affiliations:** 1 Graduate Institute of Biomedical Sciences, College of Medicine, Chang Gung University, Tao-Yuan, Taiwan; 2 Institute of Molecular Medicine, College of Medicine, National Taiwan University, Taipei, Taiwan; 3 Department of Cell and Molecular Biology, College of Medicine, Chang Gung University, Tao-Yuan, Taiwan; 4 Molecular Medicine Research Center, Chang Gung University, Tao-Yuan, Taiwan; Institute of Molecular and Cell Biology, Biopolis, UNITED STATES

## Abstract

The small GTPase Arf-like protein 1 (Arl1) is well known for its role in intracellular vesicular transport at the *trans*-Golgi network (TGN). In this study, we used differential affinity chromatography combined with mass spectrometry to identify Arf-interacting protein 1b (arfaptin-1b) as an Arl1-interacting protein and characterized a novel function for arfaptin-1 (including the arfaptin-1a and 1b isoforms) in Arl1-mediated retrograde transport. Using a Shiga-toxin subunit B (STxB) transportation assay, we demonstrated that knockdown of arfaptin-1 accelerated the retrograde transport of STxB from the endosome to the Golgi apparatus, whereas Arl1 knockdown inhibited STxB transport compared with control cells. Arfaptin-1 overexpression, but not an Arl1 binding-defective mutant (arfaptin-1b-F317A), consistently inhibited STxB transport. Exogenous arfaptin-1 expression did not interfere with the localization of the Arl1-interacting proteins golgin-97 and golgin-245 to the TGN and vice versa. Moreover, we found that the N-terminal region of arfaptin-1 was involved in the regulation of retrograde transport. Our results show that arfaptin-1 acts as a negative regulator in Arl1-mediated retrograde transport and suggest that different functional complexes containing Arl1 form in distinct microdomains and are responsible for different functions.

## Introduction

The ADP-ribosylation factors (ARFs) are members of a small GTPase family of the Ras superfamily that are involved in membrane transport regulation, organelle integrity maintenance, membrane lipid modification, cytoskeletal dynamics, and signal transduction [1]. Arf-like proteins (Arls) share 40–60% sequence identity [2], and more than twenty Arls have been identified in humans [3]. In yeast, Arl1 controls intracellular ion levels [4,5] and regulates endosomal transport and cell proliferation [6]. Arl1 is also involved in the transport of at least one GPI-anchored protein from the late Golgi to the plasma membrane [7]. In mammalian cells, Arl1 localizes to the *trans*-Golgi network (TGN) and functions in vesicular trafficking [8,9]. Arl1 regulates vesicle-mediated endosome-to-TGN transport by recruiting effectors such as the GRIP (golgin-97/RanBP2α/Imh1p/p230) domain-containing proteins golgin-97 and golgin-245 to the TGN [10,11]. However, the detailed molecular mechanism of how ARL1 mediates vesicular trafficking remains unknown.

Several Arl1 effectors have been identified, including golgin-245, golgin-97, MKLP1, SCOCO, pericentrin, the δ-subunit of phosphodiesterase (PDEδ), arfaptin-1a, arfaptin-2, BIG1 and BIG2 [1,12,13]. Among these effectors, two arfaptin homology (AH) domain-containing proteins, arfaptin-1a and arfaptin-2, were initially identified as Arf3-interacting proteins [14]. The sequence identity between arfaptin-1a (short form) and arfaptin-2 is 60%. Arfaptin-2 specifically interacts with GTP-bound Arf1, Arf6, Arl1 and Rac1 as well as mediates cross-talk between the Rac and Arf signaling pathways [2,8,15,16]. Arfaptin-2 is also involved in regulating huntingtin protein aggregation, possibly by impairing proteasome function [17,18]. In 2001, Venter *et al*. published the sequence of arfaptin-1b, which is the long isoform of arfaptin-1 and has an insertion (residues 69–100) that is absent from arfaptin-1a [19]. Arfaptin-1a localizes to the Golgi complex via interaction with Arl1; however, the molecular function of arfaptin-1 (both the arfaptin-1a and the arfaptin-1b isoforms) has been only partially characterized [13,20,21].

In this study, using differential affinity chromatography combined with mass spectrometry, we show that arfaptin-1b specifically interacts with GTP-bound Arl1 (Arl1QL). By assessing the intracellular transport, protein-protein interactions and subcellular localization of arfaptin-1, we show that arfaptin-1 negatively regulates Arl1-mediated retrograde transport.

## Results

### Identification of Arfaptin-1b as an Arl1-interacting Protein

To gain a better understanding of the essential role that Arl1 plays *in vivo*, we isolated Arl1 protein complexes from HeLa cells by differential affinity chromatography using immobilized GST-Arl1 (Arl1QL, the putative active form of Arl1; Arl1TN, the dominant-negative form of Arl1). The protein components were identified by tandem mass spectrometry, as shown in S1 Fig. We identified 38 unique proteins that specifically interacted with GTP-bound Arl1. Detailed information on these 38 Arl1QL interaction proteins, including the probability of identification, mass score and peptide information, is shown in S1 Table. Notably, arfaptin-1 was detected in two distinct gel bands with different molecular weights. One unique peptide belonging to arfaptin-1b (LAQQGSDLIVPAGGQR) had 95% peptide identification probability (S1 Table). This isoform was ubiquitously expressed in human cancer cells, as it can be detected in 13 cancer cell lines derived from 12 different types of human organs by western blotting (S2 Fig.). We confirmed the interaction between Arl1 and its interaction partners by western blotting and a yeast two-hybrid assay (S3 Fig.). As expected, we found that Arl1QL, but not Arl1TN, interacted with arfaptin-1a, arfaptin-1b, arfaptin-2, golgin-97 and golgin-245 (S3A Fig.). Interestingly, we also found that the interaction between arfaptin-1 and Arl1QL was stronger than that between arfaptin-1 and Arf1QL. Taken together, these results show that arfaptin-1b interacts with Arl1 in a GTP-dependent manner.

### Arfaptin-1 Regulates the Retrograde Transport of STxB from Endosomes to the Golgi Apparatus

**Fig 1:**
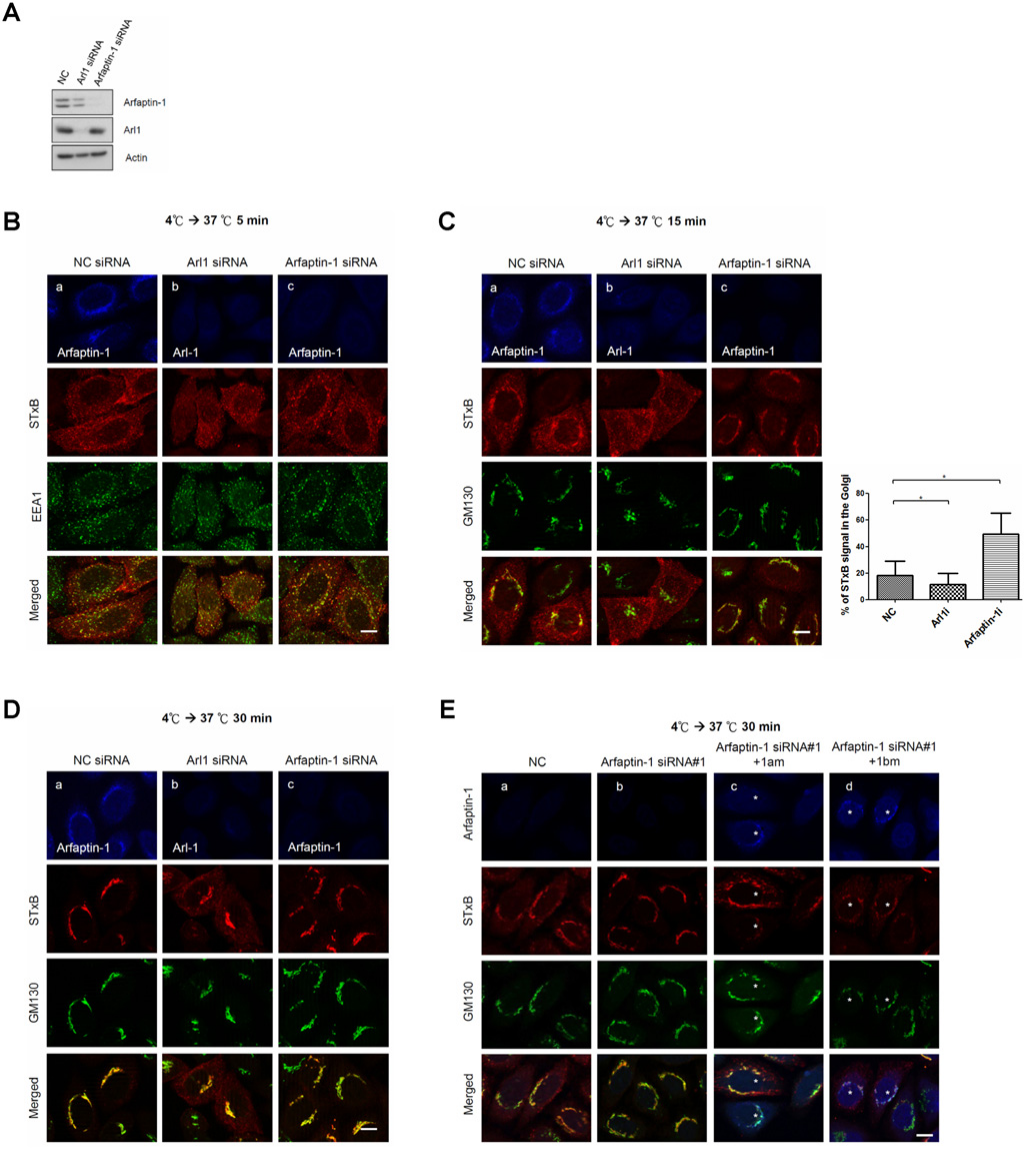
Arfaptin-1 regulates STxB transport from endosomes to the Golgi apparatus. (A) HeLa cells were transfected with control siRNA or siRNA specific for Arl1 or arfaptin-1, as indicated. After 48 h, the cells were subjected to western blotting with anti-arfaptin-1 and anti-Arl1 antibodies, respectively. Actin was used as the internal control. Simultaneously, the knockdown cells were incubated with Cy3-conjugated STxB at 4°C for 20 min and shifted to 37°C for 5 min (B), 15 min (C) and 30 min (D), followed by immunofluorescence staining with anti-EEA1, anti-GM130, anti-Arl1 and anti-arfaptin-1 antibodies as indicated. The intensity and area of STxB (red) and GM130 (green) signals were quantified as described in the Materials and Methods. The percentage of STxB signal in the Golgi was calculated using the following formula: % of STxB signal in the Golgi = total intensity of co-localization of STxB and GM130/total intensity of STxB. The results are presented as the means±SDs; *p*<0.05 indicates significance, as assessed by one-way ANOVA. (E) Exogenous expression of siRNA-resistant arfaptin-1 in arfaptin-1-knockdown cells rescued the kinetics of STxB transport. HeLa cells were transfected with control siRNA or siRNA specific for arfaptin-1. After 24 h, the cells were transfected with siRNA-resistant arfaptin-1 (arfaptin-1am-myc or arfaptin-1bm-myc) for an additional 24 h, and a STxB transport assay with incubation at 37°C for 30 min was conducted. The cells were fixed and stained with anti-GM130 and anti-myc antibodies as indicated. Scale bars, 10 μm. Asterisks indicate siRNA-resistant arfaptin-1-expressing cells.

It has been previously shown that depletion of Arl1 or golgin-97 blocks the retrograde transport of STxB from endosomes to the TGN [10,22]. We therefore examined whether arfaptin-1b is also involved in STxB endocytosis using a siRNA knockdown approach. Western blotting and immunofluorescence staining (Fig. 1A) first demonstrated the significant and homogenous reduction of arfaptin-1 and Arl1 in knockdown cells. The expression levels of arfaptin-1 and Arl1 were reduced to 16% and 21% in arfaptin-1- and Arl1-knockdown cells, respectively. Furthermore, immunofluorescence staining revealed that the transfection of siRNA caused the homogenous depletion of arafptin-1 and Arl1 in knockdown cells (data not shown). We next assessed the kinetics of STxB transport from the plasma membrane to the TGN in HeLa cells. STxB was bound to the plasma membrane at 4°C; transport to early endosomes, recycling endosomes and the TGN was activated by incubation at 37°C for 5, 15 and 30 min, respectively (S4 Fig.). As shown in Fig. 1B, after 5 min at 37°C, internalized STxB partially co-localized with EEA1 in Arl1- and arfaptin-1-knockdown cells. Arl1 knockdown inhibited STxB transport, as shown by the punctate pattern of STxB in the endosomes of Arl1-knockdown cells after 15 min or 30 min at 37°C (Fig. 1C–b and Fig. 1D–b). Unexpectedly, significant fractions of STxB were transported to the Golgi in arfaptin-1-knockdown cells after 15 min at 37°C, suggesting that the kinetics of STxB transport were accelerated in arfaptin-1-knockdown cells (Fig. 1C–c). After 30 min at 37°C, STxB co-localized extensively with GM130, suggesting that STxB was continually transported to the Golgi in arfaptin-1-knockdown cells (Fig. 1D–c). To rule out any off-target effects from the gene knockdown, we used two oligos with unique siRNA sequences to separately knock down arfaptin-1 and observed a similar effect on STxB transport (S5 Fig.). Next, we expressed siRNA-resistant arfaptin-1a (1am) and arfaptin-1b (1bm) in arfaptin-1-knockdown cells, respectively. As shown in Fig.1E, exogenous expression of siRNA-resistant arfaptin-1 in arfaptin-1-knockdown cells rescued the kinetics of STxB transport. In these cells, a significant proportion of STxB localized to endosomes in a punctate pattern after 30 min at 37°C, instead of concentrating in the Golgi region, as in arfaptin-1-knockdown cells (Fig. 1E–c, d). Taken together, these results demonstrate that the loss of arfaptin-1 accelerates STxB transport from endosomes to the Golgi apparatus, suggesting that arfaptin-1 is involved in the endocytic pathway.

### Exogenous Expression of Arfaptin-1 Inhibits STxB Transport in an Arl1-dependent Manner

**Fig 2:**
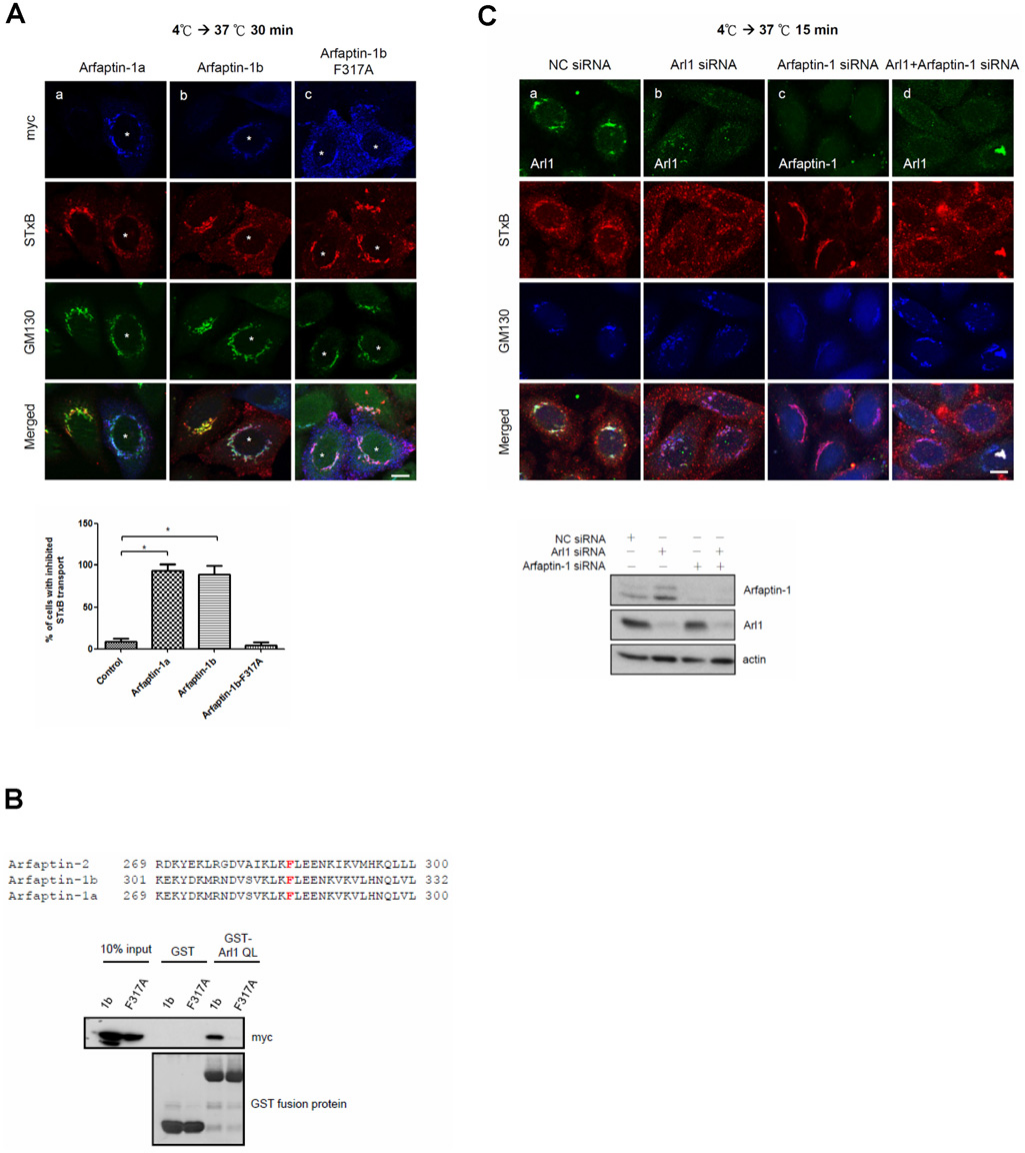
Arfaptin-1b overexpression inhibits STxB transport in an Arl1-dependent manner. (A) HeLa cells were transfected with arfaptin-1a-myc, arfaptin-1b-myc or arfaptin-1b-F317A-myc for 48 h. A STxB transport assay was conducted with incubation at 37°C for 30 min and the cells stained with anti-myc and anti-GM130 antibodies to detect exogenous arfaptin-1 and endogenous GM130, respectively. Scale bars, 10 μm. Asterisks indicate arfaptin-1a-myc- (a), arfaptin-1b-myc- (b) and arfaptin-1b-F317A-myc-expressing cells (c). The percentage of cells exhibiting inhibited STxB transport obtained from (A) was calculated (n>50 for each experiment), and the data are presented as the means±SDs; *p*<0.05 indicates significance, as determined by one-way ANOVA. (B) Arfaptin-1b-F317A-myc is unable to bind to the active form of Arl1. HeLa cells were transfected with arfaptin-1b-myc and arfaptin-1b-F317A-myc for 48 h. Recombinant GST and GST-Arl1QL were expressed in BL21 cells and immobilized on glutathione-sepharose resin. The immobilized GST fusion proteins were exchanged for GTPγS, and the proteins were incubated with lysates of transfected HeLa cells. The bound proteins were resolved on a 12.5% SDS-PAGE gel and analyzed by western blotting using an anti-myc antibody. The membrane was stained with *Coomassie Brilliant Blue* to demonstrate equal loading (lower panel). (C) Double knockdown of Arl1 and arfaptin-1 inhibits STxB transport. HeLa cells were transfected with control siRNA or siRNA specific to Arl1 or arfaptin-1 for 48 h. A STxB transport assay was performed with incubation at 37°C for 15 min, followed by immunofluorescence staining with anti-Arl1, anti-arfaptin-1 and anti-GM130 antibodies as indicated. Scale bars, 10 μm.

Because arfaptin-1 knockdown accelerated the rate of STxB transport from endosomes to the Golgi apparatus, we next questioned whether overexpression of arfaptin-1 (1a and 1b) could impair STxB transport. As shown in Fig. 2A, overexpression of arfaptin-1a or arfaptin-1b inhibited STxB transport compared with control cells, as a significant proportion of STxB was distributed in punctate in the cytoplasm rather than concentrated in the Golgi region after 30 min at 37°C. Next, we questioned whether arfaptin-1 involvement in STxB transport was dependent on Arl1. A recent study showed that the phenylalanine at residue 285 of arfaptin-2 was critical for Arl1-arfaptin-2 complex formation [23]. This phenylalanine is highly conserved in the C-terminus of arfaptins and is located at position 317 in arfaptin-1b (Fig. 2B). We therefore generated a construct expressing arfaptin-1b-F317A and confirmed that arfaptin-1b-F317A indeed lost its ability to bind to Arl1 using a GST pull-down assay (Fig. 2B). Immunofluorescence staining showed that arfaptin-1b-F317A was distributed in the cytosol and not concentrated in the Golgi apparatus. Importantly, we found that internalized STxB in cells expressing arfaptin-1b-F317A co-localized with GM130 after 30 min at 37°C, similar to the control cells (Fig. 2A–c). This result clearly demonstrates that, unlike wild-type arfaptin-1 (arfaptin-1a and arfaptin-1b), the arfaptin-1b-F317A mutant did not inhibit STxB transport (Fig. 2A). We also found that double knockdown of Arl1 and arfaptin-1 inhibited retrograde transport of STxB to an extent similar to Arl1 knockdown (Fig. 2C). Taken together, these results suggest that arfaptin-1 regulates STxB transport in an Arl1-dependent manner.

### Arfaptin-1 and GRIP Domain-containing Proteins do not Compete for Arl1 Binding at the TGN

**Fig 3:**
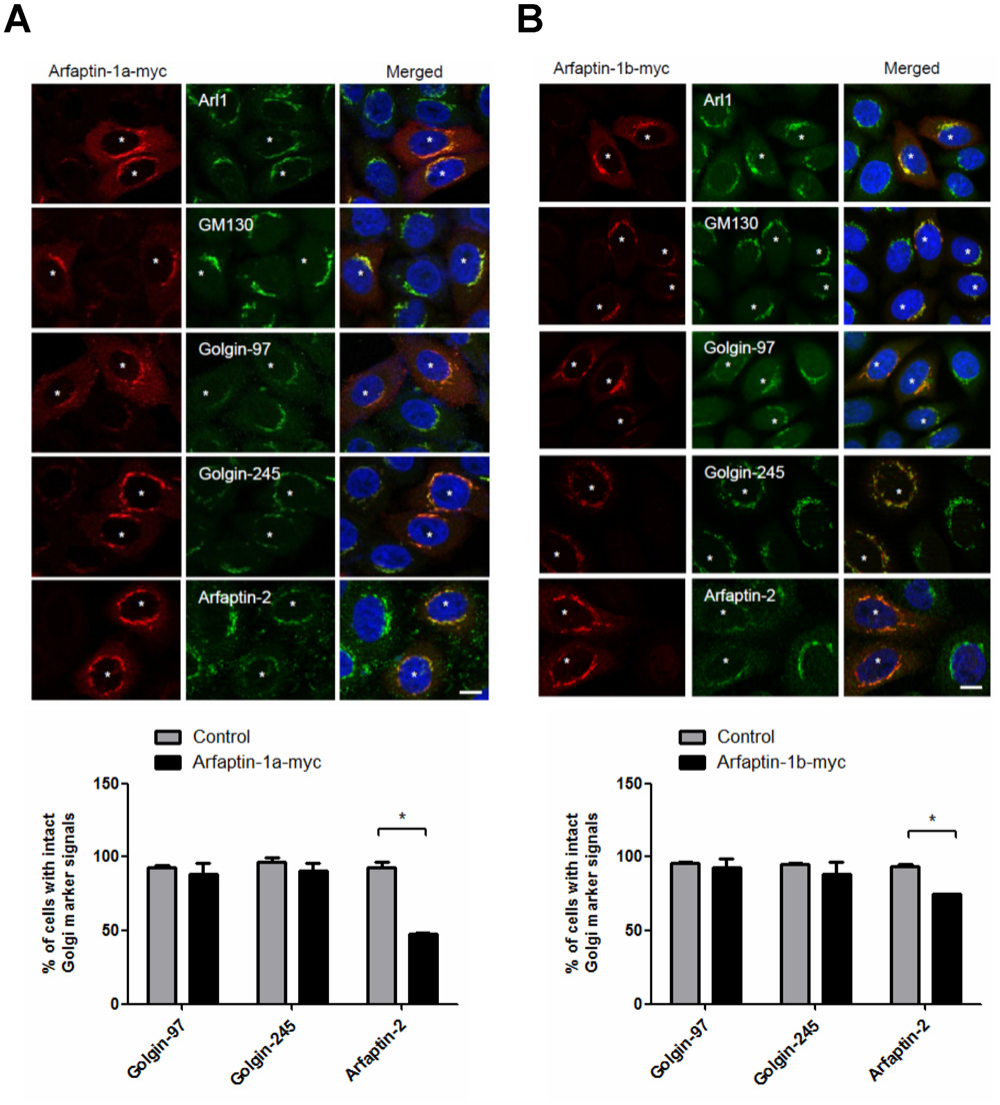
Exogenous expression of arfaptin-1 has no effect on the localization of GRIP domain-containing proteins to the Golgi. HeLa cells were transfected with arfaptin-1a-myc (A) or arfaptin-1b-myc (B) for 48 h. The cells were then stained with anti-myc and anti-Golgi marker protein antibodies (anti-Arl1, anti-GM130, anti-golgin-97, anti-golgin-245 and anti-arfaptin-2). The percentage of arfaptin-1a-myc and arfaptin-1b-myc-expressing cells (n>100 for each experiment) with intact Golgi marker signals were quantified, and the data are presented as the means±SDs; *p*<0.05 indicates statistical significance, as determined by the unpaired Student’s *t* test. Scale bars, 10 μm. Asterisks indicate arfaptin-1a-myc- (A) or arfaptin-1b-myc- (B) expressing cells.

**Fig 4:**
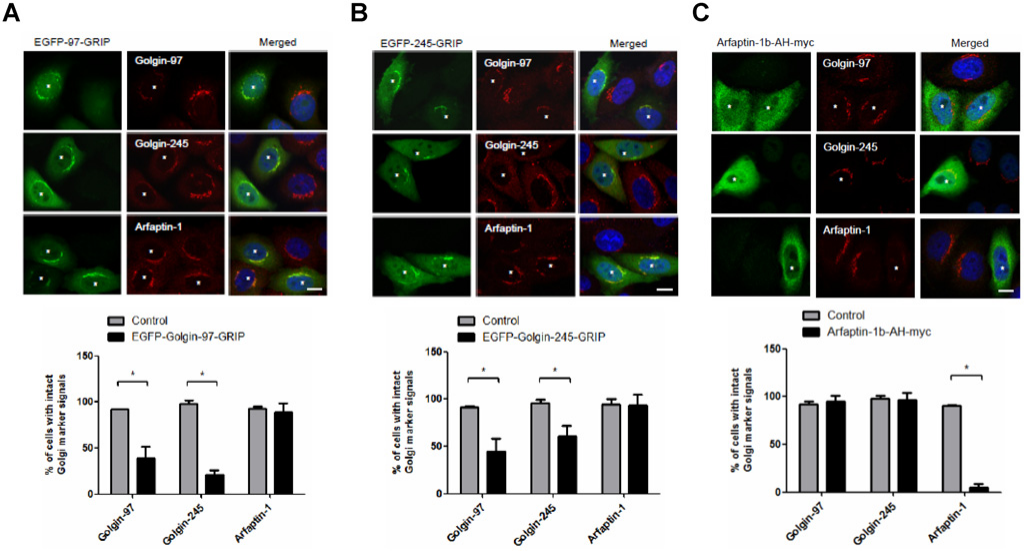
Exogenous expression of the EGFP-GRIP domain does not induce the dissociation of arfaptin-1 from the Golgi apparatus. HeLa cells were transfected with EGFP-97-GRIP (A), EGFP-245-GRIP (B) or arfaptin-1b-AH-myc (C) for 48 h, and the cells were then stained with anti-myc and anti-golgin antibodies (anti-golgin-97, anti-golgin-245 and anti-arfaptin-1). The percentage of EGFP-GRIP- or arfaptin-1b-AH-myc-expressing cells (n>50 for each experiment) with intact Golgi marker signals was quantified, and the data are presented as the means±SDs; *p*<0.05 indicates significance, as determined by the unpaired Student’s *t* test. Scale bars, 10 μm. Asterisks indicate EGFP-97-GRIP- (A), EGFP-245-GRIP- (B) or arfaptin-1-AH-myc-expressing (C) cells. It is notable that the epitope of polyclonal goat anti-arfaptin-1 (Santa Cruz, sc19246) that was used in the current study is near the N terminus of arfaptin-1 (1a and 1b); therefore, it could not be used to detect arfaptin-1b-AH-myc because the AH domain is located in the C-terminal region of afaptin-1.

GRIP domain-containing proteins, such as golgin-97 and golgin-245, interact with Arl1 at the TGN and function as tethering molecules in retrograde traffic [10,11]. In this study, we found that arfaptin-1 depletion accelerated STxB transport from endosomes to the Golgi, suggesting that arfaptin-1 and GRIP domain-containing proteins may form distinct functional complexes with Arl1. To test this possibility, we first questioned whether arfaptin-1 and GRIP domain-containing proteins compete to form complexes at the TGN. Immunofluorescence analysis showed that overexpression of arfaptin-1 (arfaptin-1a and arfaptin-1b) did not induce the dissociation of endogenous golgin-245 and golgin-97 from the Golgi (Fig. 3). Nevertheless, the amount of arfaptin-2 localized to the Golgi was significantly lower in arfaptin-1-expressing cells. The distributions of Arl1 and GM130 were identical in arfaptin-1-expressing cells and control cells, suggesting that the Golgi structure was intact in arfaptin-1-expressing cells. Next, we overexpressed EGFP-golgin 97-GRIP and EGFP-golgin 245-GRIP in HeLa cells. As shown in Fig. 4A and Fig. 4B, overexpression of the GRIP domain of golgin-97 or golgin-245 had no effect on the localization of endogenous arfaptin-1 to the Golgi. In contrast, these cells showed significant dissociation of endogenous golgin-97 and golgin-245. Taken together, these results demonstrate that arfaptin-1 and GRIP domain-containing proteins do not compete for Arl1 binding at the TGN, suggesting that arfaptin-1 and GRIP domain-containing proteins may form distinct functional complexes with Arl1.

### The N-terminal Region of Arfaptin-1 Is Required for Maintaining its Golgi Localization and for Regulating Retrograde Transport

The AH domain, also known as the Bin/Amphiphysin/Rvs (BAR) domain, is located in the C-terminus of arfaptin-2 and arfaptin-1 and is responsible for binding to Arl1 [13,23]. However, overexpression of the AH domain of arfaptin-1b did not interfere with the localization of endogenous golgin-97 and golgin-245 to the Golgi (Fig. 4C), suggesting that arfaptin-1 and GRIP domain-containing proteins form different functional complexes with Arl1 at the TGN. We suspected that arfaptin-1 might contain elements other than the AH domain to mediate Arl1-arafptin-1 complex formation at the TGN. Using a yeast two-hybrid assay, we determined that the N-terminal region of arfaptin-1 (arfaptin-1a-S1, residues 1–116; arfaptin-1b-L1, residues 1–148) did not interact with Arl1QL (S3B Fig.). As expected, the C-terminal region of arfaptin-1b (arfaptin-1b-L23, residues 143–373) was sufficient for Arl1 binding. When the C-terminal region was separated into two fragments (arfaptin-1b-L2 and arfaptin-1b-L3), neither fragment was able to bind to Arl1. Unexpectedly, immunofluorescence staining showed that exogenous arfaptin-1b-AH (residues 153–353 of arfaptin-1b) was diffuse throughout the cells rather than concentrated at the TGN (Fig. 4C). Endogenous arfaptin-1 did not localize to the Golgi in arfaptin-1b-AH-expressing cells, suggesting that the interaction between the AH domain and Arl1 is required to target arfaptin-1 to the Golgi but is insufficient to maintain arfaptin-1 in the Golgi.

**Fig 5:**
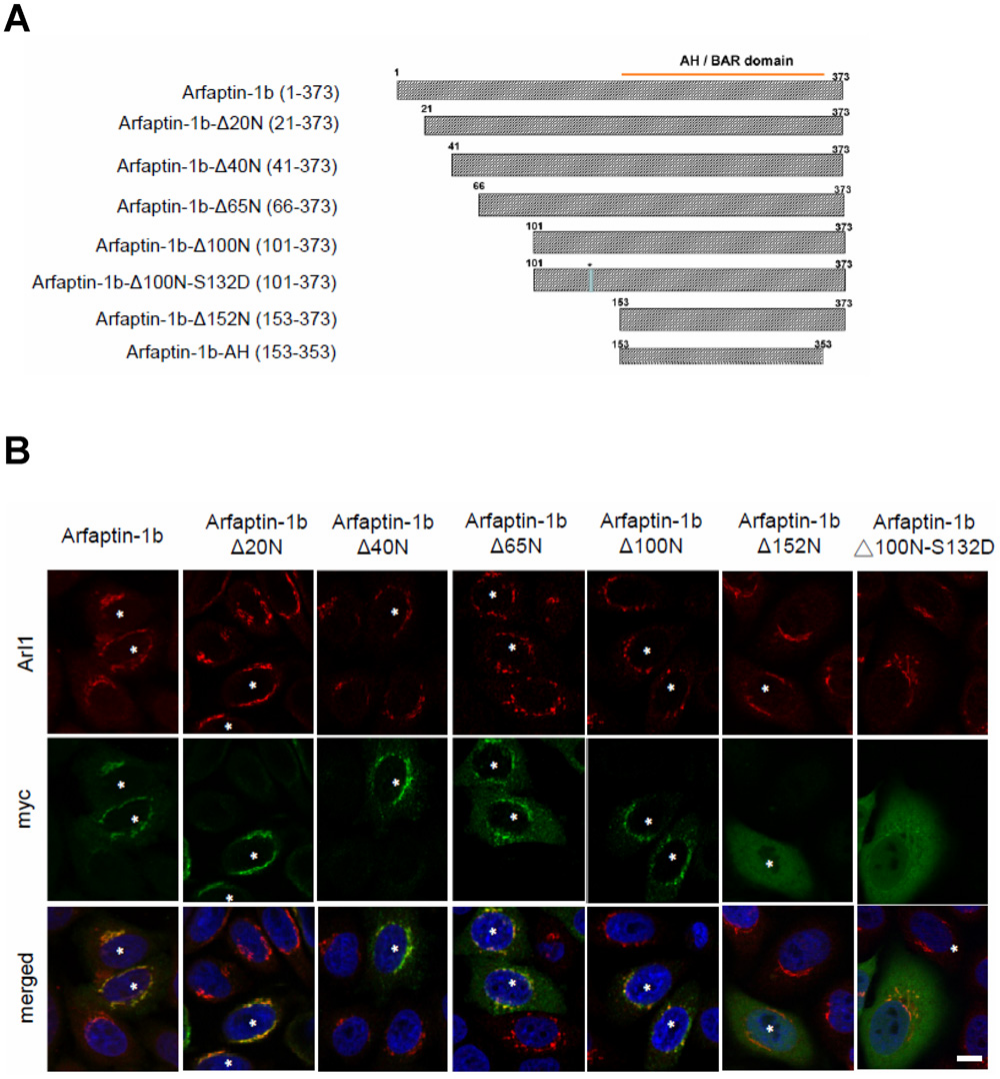
The N-terminal region of arfaptin-1 is required for localization to the Golgi. (A) Schematic showing the arfaptin-1 N-terminal truncations and mutations used in this study. (B) HeLa cells were transfected with wild-type or truncated arfaptin-1b-myc constructs for 48 h. The cells were then stained with anti-myc and anti-Arl1 antibodies. Scale bars, 10 μm. Asterisks indicate cells expressing truncated or mutated arfaptin-1-myc constructs.

Notably, the C-terminus of arfaptin-1 contains the AH/BAR-domain but does not contain any membrane interaction modules, such as the pleckstrin homology (PH) domain or the Phox homology (PX) domain, to sense and/or induce curvature in the N-terminus [24]. Therefore, we hypothesized that the N-terminal region of arfaptin-1 may play a role in membrane association and thereby contribute to maintaining localization within the Golgi. We predicted the secondary structure of arfaptin-1b using the Protein Homology/analogY Recognition Engine (http://www.sbg.bio.ic.ac.uk/~phyre/), and based on the prediction, we constructed five truncated arfaptin-1b mutants and expressed them in HeLa cells (Fig. 5). Immunofluorescence staining showed that exogenous expression of the N-terminally truncated arfaptin-1b mutants (i.e., expression of arfaptin-1b-Δ40N, Δ65N and Δ100N) led to sequentially increasing proportions of arfaptin-1 distributed throughout the cytosol, while a portion of the exogenously expressed protein remained co-localized with Arl1 in the Golgi apparatus. Importantly, without the first 152 residues (arfaptin-1b-Δ 152), arfaptin-1b would dramatically dissociate from TGN (Fig. 5B). These results suggested that the N-terminal region of arfaptin-1 may contribute to maintaining arfaptin-1 localization in the Golgi, and residues 100 to 153 are the most critical region for this function. This conclusion is consistent with a recent study by Gehart *et al*., which reported that protein kinase D (PKD) mediates the phosphorylation of arfaptin-1b at serine 132 and causes arfaptin-1b mis-localization to the Golgi [21]. In this study, we used the arfaptin-1b-Δ 100N-S132D construct to mimic the highly phosphorylated status of arfaptin-1b and found that arfaptin-1b-Δ100N-S132D was distributed uniformly in cells, similar to arfaptin-1b-Δ152N (Fig. 5B). Taken together, these results showed that the N-terminal region of arfaptin-1 is important for localization to the Golgi, and serine 132 is critical for this function.

**Fig 6:**
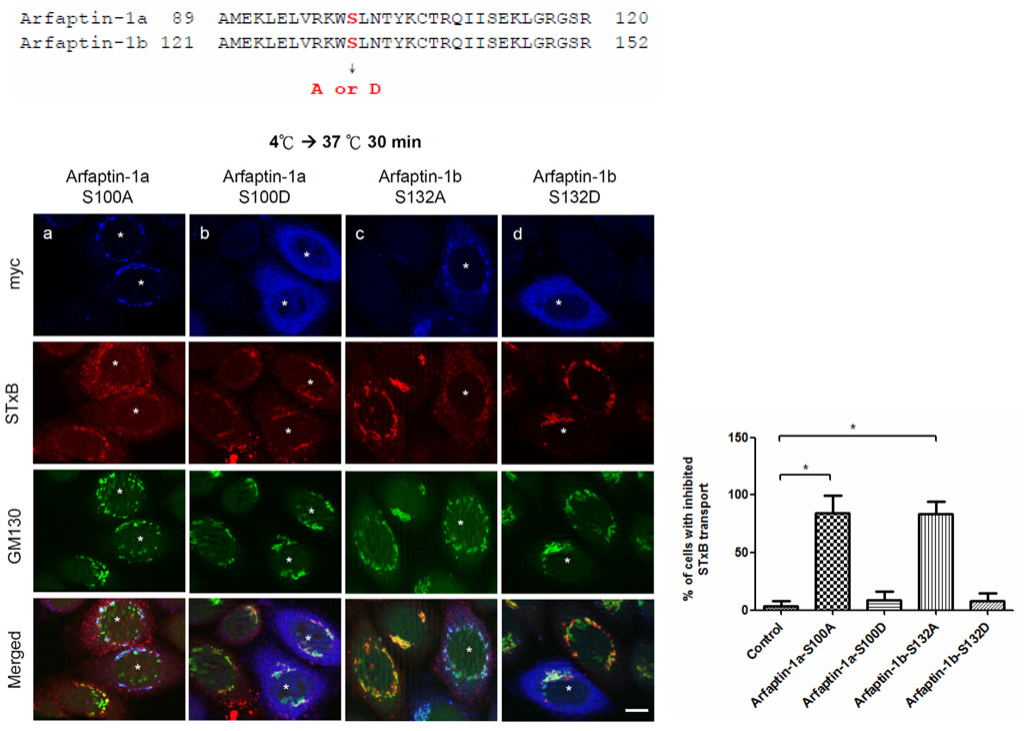
The effect of arfaptin-1b serine 132 phosphorylation on STxB endocytic transport. HeLa cells were transfected with arfaptin-1a-S100A, arfaptin-1a-S100D, arfaptin-1b-S132A, or arfaptin-1b-S132D for 48 h. A STxB transport assay was conducted with incubation at 37°C for 30 min, followed by staining with anti-myc and anti-GM130 antibodies. Scale bars, 10 μm. Asterisks indicate the exogenous expression of myc-tagged arfaptin-1 constructs. The percentage of cells exhibiting inhibited STxB transport was quantified (n>50 for each experiment), and the data are presented as the means±SDs; *p*<0.05 indicates significance, as determined by one-way ANOVA.

Arfaptin-1b acts as a negative regulator of Arf1-mediated vesicle budding [21]. In this study, we identified a potential function for arfaptin-1 in the endocytic pathway and confirmed the role of serine 132 in localization to the Golgi. We next questioned whether the arfaptin-1 phosphorylation status could also regulate retrograde transport of STxB. As shown in Fig. 6, similar to wild-type arfaptin-1b, arfaptin-1b-S132A inhibited STxB transport after 30 min at 37°C compared with control cells. However, STxB transport in arfaptin-1b-S132D-expressing cells was similar to that in control cells after 30 min at 37°C. Similar results were observed in cells expressing arfaptin-1a-S100A and arfaptin-1a-S100D (Fig. 6). These results suggest that PKD—mediated phosphorylation of arfaptin-1 is involved in the regulation of retrograde transport.

## Discussion

**Fig 7:**
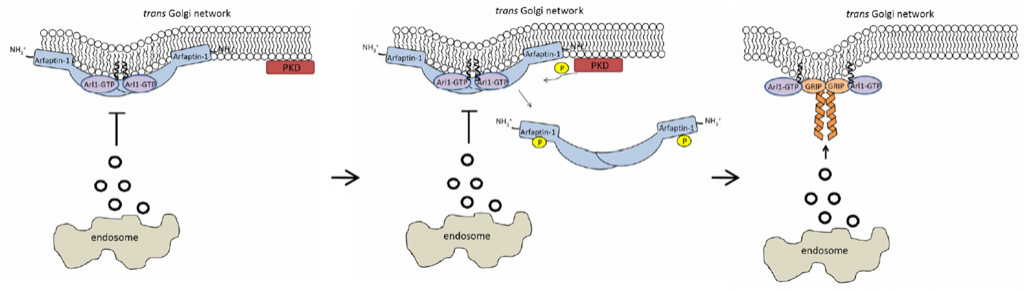
Schematic of the proposed role of arfaptin-1 in Arl1-mediated retrograde transport. Arfaptin-1 is recruited to the TGN through interaction with GTP-bound Arl1, where it inhibits vesicle transport from endosomes to the Golgi. Arfaptin-1 disassociates from GTP-bound Arl1 until protein kinase D phosphorylates arfaptin-1 at the N-terminus. The GTP-bound Arl1 then recruits GRIP domain-containing proteins to the TGN and receives the cargo proteins transported from the endosomes to the Golgi.

In this study, we found that Arl1 and arfaptin-1 have inverse effects on retrograde transport. Arl1 knockdown inhibited retrograde transport of STxB from endosomes to the Golgi, while arfaptin-1 knockdown accelerated STxB transport to the Golgi. Arfaptin-1a is a shared effector of Arf1 and Arl1 and has been reported to inhibit the Arf-dependent transport of cholera toxin ADP-ribosyltransferase, phospholipase D and VSV-G from the ER to the Golgi *in vitro* [25,26]. Recently, Gehart *et al*. reported that arfaptin-1 binds to the active form of Arf1 and prevents Arf1-mediated membrane fission [21]. Consistent with their observations, our data show that arfaptin-1 negatively regulates Arl1-mediated retrograde transport and also suggests that arfaptin-1 and GRIP domain-containing proteins may form different functional complexes with GTP-bound Arl1 at the TGN. Our results are consistent with previous reports showing that TGN has multiple biochemically and functionally distinct subdomains, each of which participates in sorting and transport in this dynamic compartment. [27,28]. Therefore, we hypothesized that the Arl1-arfaptin-1 complex may affect the structure and function of a TGN subdomain, thus regulating Arl1-Golgin-mediated retrograde transport (Fig. 7).

Recently, Boucrot *et al*. used a new *in vitro* quantitative vesiculation assay to reveal an antagonistic relationship between amphipathic helices and N-BAR domain scaffolds in fission. The authors proposed that shallow hydrophobic insertions and crescent-shaped BAR scaffolds promote membrane curvature [29]. Tsai *et al*. reported a unique function for Arl1p in membrane remodeling, in which activated Arl1p promotes the spatial modulation of membrane organization at the TGN through interactions with Arf-GEF Gea2p and the flippase Drs2p. That study also showed that the Arl1p-Drs2p-Gea2p complex is specifically required for recruiting the GRIP domain-containing protein Imh1p to the Golgi [30,31]. These studies suggest that Arl1, together with its effectors and a flippase, induces membrane curvature at the TGN, providing a platform for coat protein, coat accessory protein or tethering protein binding, and regulating the retrieval of selected cargo proteins from endosomes to the TGN. Although future work is needed to determine how arfaptin-1 function in vesicle transport is regulated, our study provides new insight into how arfaptin-1 modulates retrograde transport from endosomes to the Golgi.

Structural analysis of Arl1-GTP in complex with the GRIP domain also demonstrated that the GRIP domain forms a homodimer that interacts with two Arl1-GTP molecules [32,33]. The N-terminal coiled-coil region preceding the GRIP domain of Imh1p facilitates homodimerization in yeast [34]. Gehart *et al*. demonstrated that PKD phosphorylation of arfaptin-1 at serine 132 induces its release from ARF, resulting in the de-inhibition of fission [21]. In this study, we confirmed the role of the N-terminus of arfaptin-1 in localization to the Golgi and retrograde transport (Fig. 5B and Fig. 6). In addition, Cruz-Garcia and his colleagues found that replacing tryptophan 99 with alanine reduced arfaptin1a binding to phosphatidylinositol 4-phosphate-containing liposomes by 80% and abolished Golgi targeting [20]. Taken together, the results from these studies support the idea that the N-terminal region of arfaptin-1 plays multiple roles in Golgi localization, Golgi membrane association, homodimerization, vesicle trafficking and the response to kinase signaling.

Structural analyses have shown that the switch II region of GTP-bound Arl1 specifically interacts with the GRIP domain of golgin-245 and the BAR domain of arfaptin-2 [23,32,33]. In this study, we introduced a myc tag to the C-terminus of arfaptin-1 to identify the spatial diversity of Arll complexes. This myc tag had no effect on the localization of GRIP domain-containing proteins to the Golgi, and expression of the GRIP domain of golgin-245 or golgin-97 had no effect on the localization of endogenous arfaptin-1 to the TGN. These results are in contrast to a previous report that exogenous expression of EGFP-arfaptin-1 interferes with the localization of golgin-245 and golgin-97 to the TGN [13]. This discrepancy could be due to differences in the tag used, as we obtained similar results to Man *et al*. when we overexpressed EGFP-arfaptin-1 in HeLa cells (S6 Fig.). To test this possibility, we further constructed arfaptin-1 (1a and 1b) without any tag (tag-free) and expressed it in HeLa cells (S7 Fig.). Similar to the results obtained for myc-tagged arfaptin-1-expressing cells (Fig. 3), expression of tag-free arfaptin-1 (1a and 1b) did not induce the dissociation of endogenous golgin-245 and golgin-97 from the Golgi. Considering that expression of the GRIP domain of golgin-97 or golgin-245 also had no effect on the localization of endogenous arfaptin-1 to the Golgi (Fig. 4A and 4B), we concluded that arfaptin-1 and GRIP domain-containing proteins did not compete for Arl1 binding in the TGN. Although Man *et al*. previously reported that knocking down arfaptins had no apparent effect on STxB transport [13], our study using cells labeled with Cy3-conjugated STxB at 4°C for 20 min (Fig. 1C) or 50 min (S8 Fig.) followed by incubation at 37°C for 15 min showed that arfaptin-1 knockdown led to accelerated STxB transport from endosomes to the Golgi apparatus. This discrepancy could be due to the different time course used to assess the retrograde transport of STxB and highlights the precise regulation involved in intracellular vesicle transport.

In conclusion, our data suggest that, while arfaptin-1 and GRIP domain-containing proteins both specifically interact with GTP-bound Arl1, these interactions are likely independent. We hypothesize that different functional complexes containing Arl1 form in distinct microdomains and are responsible for different functions. When and how do these functional complexes form? Is any specific signaling pathway responsible for the formation of these different complexes? More research is needed to answer the questions regarding the selective cargo transport that is regulated by functional Arl1 complexes.

## Materials and Methods

### Cell culture and Transient Transfections

HeLa cells were cultured in Dulbecco’s Modified Eagle’s Medium (*Gibco*, Invitrogen, Carlsbad, CA, USA) supplemented with 10% fetal bovine serum plus 100 units/ml penicillin and streptomycin at 37°C in a humidified 95% air/5% CO_2_ atmosphere. The cells were transfected with Lipofectamine 2000 (Invitrogen, Carlsbad, CA, USA) according to the manufacturer’s instructions. At 48 h after transfection, the cells were fixed or harvested for further analysis.

### Antibodies

To generate the anti-human Arl1 polyclonal antibody, a New Zealand White rabbit was immunized with a BSA-conjugated synthetic peptide corresponding to residues 130–145 of Arl1 (MEQAMTSSEMANSLGL). The anti-Arl1 antibody was purified with Arl1 peptide-conjugated Sepharose S4B (GE Healthcare, Little Chalfont, Buckinghamshire, United Kingdom). An anti-golgin-245 polyclonal antibody was generated by immunizing a New Zealand White rabbit with His-tagged golgin-245 (residues 1–271). The commercially available primary antibodies used in this study included the following: monoclonal mouse anti-p230/golgin-245 (BD Biosciences, San Jose, CA, USA); monoclonal mouse anti-GM130 (BD Biosciences); monoclonal mouse anti-golgin-97 (Molecular Probes, Eugene, OR, USA); monoclonal mouse anti-myc (Millipore, Billerica, MA, USA); polyclonal rabbit-myc (Cell Signaling Technology, Danvers, MA, USA); polyclonal goat anti-arfaptin-1 (Santa Cruz Biotechnology, Santa Cruz, CA, USA); and anti-arfaptin-2 (Santa Cruz Biotechnology). The secondary antibodies used for western blotting included HRP-conjugated goat anti-rabbit and goat anti-mouse IgG antibodies purchased from GE Healthcare. Immunofluorescence staining patterns were detected using Alexa Fluor 594-, Alexa Fluor 488- or Alexa Fluor 350-conjugated secondary antibodies purchased from Molecular Probes.

### Plasmid Constructs

The plasmids encoding Arl1 and arfaptin-1 were constructed using PCR. Using a two-step PCR procedure, we replaced the codon for Gln71 with the codon for Leu to generate a construct encoding Arl1QL. To generate the glutathione-S-transferase (GST) fusion construct for expression in *E*. *coli*, we subcloned the fragment encoding Arl1QL into the pGEX4T vector (GE Healthcare) via the EcoRI site. The plasmids encoding siRNA-resistant arfaptin-1a (arfaptin-1am) and siRNA-resistant arfaptin-1b (arfaptin-1bm) were generated using the QuikChange Multi Site-Directed Mutagenesis Kit (Agilent Technologies, Stratagene Products Division, La Jolla, CA, USA), according to the manufacturer’s instructions. The plasmids encoding arfaptin-1a-S100A, arfaptin-1a-S100D, arfaptin-1b-S132A, arfaptin-1b-S132D and arfaptin-1b-F317A were generated using a two-step PCR procedure. To generate the myc-tagged construct for expression in HeLa cells, full length arfaptin-1a and arfaptin-1b, siRNA-resistant arfaptin-1a and arfaptin-1b, truncated arfaptin-1b, and mutated arfaptin-1b were subcloned into pcDNA3.1A (Invitrogen). The sequences encoding golgin-97-GRIP and golgin-245-GRIP were amplified by PCR and subcloned into pEGFP-C2 (Clontech Laboratories, Inc., Mountain View, CA USA). The sequences of all of the primers used for plasmid construction are shown in S2 Table.

### RNA Interference Experiments

Small interfering RNA (siRNA) duplexes targeting human Arl1, arfaptin-1 and arfaptin-2 were synthesized and developed using the highly effective Stealth RNAi siRNA technology from Invitrogen. The siRNA sequences used in this study are shown in S3 Table. For gene knockdown, HeLa cells were transfected with siRNA using Lipofectamin RNAiMAX transfection reagent (Invitrogen, Grand Island, NY, USA) according to the manufacturer’s instructions. At 48 h after transfection, the cells were lysed for western blotting or stained for immunofluorescence assays.

### Immunofluorescence Staining and Image Quantification

The transfected cells were grown on coverslips in 12-well culture plates. After 48-h incubation, the cells were fixed with 4% formaldehyde and permeabilized with a permeabilization buffer (0.1% Triton X-100 and 0.05% SDS in PBS). The cells were blocked in blocking solution (0.1% saponin and 0.2% BSA in PBS) and incubated with primary antibodies. After washing, the cells were incubated with Alexa fluor-conjugated secondary antibodies (Molecular Probes) or/and Hoechst 33258 (Molecular Probes) in blocking solution. Following a second wash with PBS, the cells were mounted with 90% glycerol in PBS containing 1 mg/ml of ρ-phenylenediamine. Images were acquired using a Zeiss Apotome fluorescence microscope and Axio vision Rel 4.8 software (Carl Zeiss, Gottingen, Germany). The immunofluorescence signals were acquired using Axio vision Rel 4.8 software (Carl Zeiss). The co-localization analysis was performed using the *MetaMorph Software suite (version*, 7.1.3., Molecular Devices, LLC, Sunnyvale, CA, USA). Briefly, the area, average intensity and total intensity (area × average intensity) of the fluorescence signals for GM130 and STxB and for co-localization of these two molecules were quantitatively obtained to measure co-localization. The percentage of STxB signal in the Golgi was calculated using the following formula: % STxB signal in the Golgi = total intensity of co-localization of STxB and GM130/total intensity of STxB.

### STxB Transportation Assay

HeLa cells were grown on coverslips in 12-well culture plates. Cy3-conjugated STxB was prepared as previously described [35] and added to the culture medium at 4°C for 20 min to allow STxB to bind to the plasma membrane. After washing with ice-cold PBS, we initiated internalization by shifting the incubation temperature to 37°C. The cells were fixed at different time points (0, 5, 15 or 30 min), followed by immunofluorescence staining. The organelle-specific protein markers EEA1, transferrin receptor and GM130 were used to identify early endosomes, recycling endosomes and the Golgi apparatus, respectively.

### *In vitro* Pull-down Assay

Recombinant GST and GST-Arl1QL were expressed in BL21 cells and immobilized on glutathione-sepharose resin (GE Healthcare). The immobilized GST fusion proteins were exchanged for GTPγS (Roche, Mannheim, Germany) according to the protocol described in Lu *et al*. [36]. Briefly, the GST fusion proteins were equilibrated in NE buffer (20 mM HEPES, pH 7.5, 100 mM NaCl, 10 mM EDTA, 5 mM MgCl_2_, and 1 mM DTT) with 0.1% (w/v) sodium cholate and 10 μM GTPγS. After equilibration, the resin was incubated with NE buffer containing 1 mM GTPγS, 0.1% sodium cholate and 3 mM L-α-dimyristoylphosphatidylcholine for 1.5 h at RT. The resins were then washed and equilibrated in NS buffer (20 mM HEPES, pH 7.5, 100 mM NaCl, 5 mM MgCl_2_, and 1 mM DTT) with 10 μM GTPγS. HeLa cells were transfected with arfaptin-1b-myc or arfaptin-1b-F317A-myc for 48 h. The transfected cells were then lysed, and the lysates were suspended in NS buffer and precleared by incubating with immobilized GST resin (1 mg HeLa cell lysates/10 μg GST proteins) at 4°C for 2 h. The resulting unbound fractions were collected and incubated with immobilized GST fusion proteins (2.5 mg HeLa cell lysates/5 μg GST fusion proteins) at 4°C overnight. After washing, the bound proteins were resolved on a 12.5% SDS-PAGE gel and analyzed by western blot assays.

## Supporting Information

S1 Table Protein list of 38 Arl1QL-specific interaction proteins. (XLS)

S2 Table The primer sequences used for plasmid constructions. (XLS)

S3 Table The siRNA sequences used in gene knockdown experiments. (XLS)

S1 Fig Strategy used to identify Arl1-interacting proteins. Schematic of the workflow used to identify Arl1-interacting proteins. The proteomics-based approaches that were used included differential affinity chromatography with immobilized GST-tagged Arl1 (Arl1QL, Arl1 TN and GST control), one-dimensional SDS-PAGE combined with nano-LC-MS/MS, database searches, validation of MS/MS-based peptides and protein identification using Scaffold proteome software. Arl1QL was used as a putative active form of Arl1, whereas Arl1TN was used as a dominant-negative form (inactive form) of Arl1. The plasmid constructions for the GST fusion proteins are described in the S1 document.
(PDF)

S2 Fig Arfaptin-1 is ubiquitously expressed in human cancer cell lines. Total protein extracts prepared from cancer cell lines (30 μg per lane) were analyzed by western blotting using anti-arfaptin-1 antibody. Actin was used as an internal control
(PDF)

S3 Fig Verification of Arl1-interacting proteins by western blotting and yeast two-hybrid assays. (A) Differential affinity chromatography followed by western blotting showed that endogenous golgin-245, golgin-97 and arfaptin-2 and two isoforms of arfaptin-1 interact specifically with Arl1QL. Recombinant GST-Arl1QL, GST-Arl1TN, GST-Arf1QL, GST-Arf1TN and GST were produced by BL21 cells and immobilized on glutathione-sepharose resin. The immobilized GST fusion proteins were incubated with GTPγS and GDP for the QL and TN proteins, respectively. HeLa lysates (2.5 mg) were then incubated with 5 μg of the GST fusion proteins, and the bound proteins were resolved on a 12.5% SDS-PAGE and analyzed by western blotting. The membrane was stained with *Coomassie Brilliant Blue* to demonstrate equal loading (lower panel), and image quantification revealed that the level of arfaptin-1 bound to Arl1QL was higher (1.84-fold change) than that to Arf1QL. (B) The C-terminal domain of arfaptin-1 (1a and 1b) interacts directly with Arl1QL in a yeast two-hybrid system. Top, diagram of arfaptin-1 and the deletion constructs. Bottom, the small GTPase constructs (Arf1QL, Arl1QL or Arl1TN) fused to the LexA DNA-binding domain and the indicated arfaptin-1 constructs (arfaptin-1a, arfaptin-1b, arfaptin-1a-S1, arfaptin-1b-L1, arfaptin-1b-L2, arfaptin-1b-L3 or arfaptin-1b-L23) fused to the GAL4-activation domain were co-transformed into yeast strain L40. The resulting transformants were plated, and the colonies were screened for histidine auxotrophy. Lamin was used as a negative control.
(PDF)

S4 Fig The kinetics of STxB transportation. HeLa cells were grown on coverslips in a 12-well culture plate for 24 h. Cy3-conjugated STxB was then added to the cultured cells and allowed to bind to the plasma membrane at 4°C for 20 min. The cells were then shifted to 37°C for 5 min, 15 min or 30 min and then fixed and stained with anti-EEA1, anti-TfR and anti-GM130 antibodies. The images were acquired using a Zeiss Apotome fluorescence microscope and Axio vision Rel 4.8 software (Carl Zeiss, Gottingen, Germany). Scale bars, 10 μm.
(PDF)

S5 Fig Arfaptin-1 knockdown accelerates STxB transport from endosomes to the Golgi apparatus. HeLa cells were transfected with two unique arfaptin-1 siRNA sequences (oligo #1 or oligo #2) or a control siRNA. After 48 h, the cells were incubated with Cy3-conjugated STxB at 4°C for 20 min and then shifted to 37°C for 15 min. The cells were then fixed and stained with an anti-GM130 antibody. The images were acquired using a Zeiss Apotome fluorescence microscope and Axio vision Rel 4.8 software (Carl Zeiss, Gottingen, Germany). The intensity and area of the STxB (red) and GM130 (green) signals were quantified, and the percentage of the STxB signal in the Golgi was calculated using the following formula: % of STxB signal in the Golgi = total intensity of co-localization of STxB and GM130/total intensity of STxB. The results are presented as the means±SDs; *p*<0.05 indicates significance, as determined by one-way ANOVA. Scale bars, 10 μm. Asterisks indicate arfaptin-1-knockdown cells.
(PDF)

S6 Fig Exogenous expression of EGFP-arfaptin-1 induces the dissociation of golgin-97 and golgin-245 from the Golgi apparatus. HeLa cells were transfected with EGFP-arfaptin-1a (A) or EGFP-arfaptin-1b (B) for 48 h and then stained with anti-golgin-97, anti-golgin-245 and anti-Arl1 antibodies. EGFP-arfaptin-1a and EGFP-arfaptin-1b-expressing cells (n>100 for each experiment) with intact Golgi marker signals were quantified. The results are presented as the means±SDs; *p*<0.05 indicates significance, as determined by an unpaired Student’s *t* test. Scale bars, 10 μm. Asterisks indicate EGFP-arfaptin-1a- (A) or EGFP-arfaptin-1b-expressing (B) cells.
(PDF)

S7 Fig Exogenous expression of arfaptin-1 without a tag has no effect on the localization of GRIP domain-containing proteins to the Golgi. (A) HeLa cells were transfected with tag-free arfaptin-1a, tag-free arfaptin-1b, arfaptin-1a-myc or arfaptin-1b-myc for 48 h, and the cell extracts were analyzed by western blotting using anti-arfaptin-1 and anti-myc as indicated. Actin was use as the internal control. Asterisks indicate endogenous arfaptin-1. (B and C) HeLa cells were transfected with tag-free arfaptin-1a or arfaptin-1b for 48 h and processed for immunofluorescence staining with anti-arfaptin-1, anti-golgin-97 and anti-golgin-245 antibodies as indicated. Arfaptin-1a and arfaptin-1b-expressing cells (n>50 for each experiment) with intact Golgi marker signals were quantified. The results are presented as the means±SDs; *p*>0.05 indicates non-significance, as determined by an unpaired Student’s *t* test. Scale bars, 10 μm. Asterisks indicate the tag-free arfaptin-1a- (B) or arfaptin-1b-expressing (C) cells.
(PDF)

S8 Fig Loss of arfaptin-1 accelerates STxB transport from endosomes to the Golgi apparatus when incubated for 50 min at 4°C. HeLa cells were transfected with control siRNA or siRNA specific for Arl1 or arfaptin-1, as indicated. After 48 h, the cells were incubated with Cy3-conjugated STxB at 4°C for 50 min and then shifted to 37°C for 15 min followed by immunofluorescence staining with anti-Arl1, anti-arfaptin-1, or anti-GM130 antibodies as indicated. The intensity and area of the STxB (red) and GM130 (green) signals were quantified (n>50), and the percentage of STxB signal in the Golgi was calculated using the following formula: % of STxB signal in the Golgi = total intensity of co-localization of STxB and GM130/total intensity of STxB. The results are presented as the means±SDs; p<0.05 indicates significance, as assessed by the unpaired Student’s *t* test.
(PDF)

S1 Document Materials and Methods for Supplemental Results. (DOC)
