# Supplementary material for: Arfaptin-1 Negatively Regulates Arl1-Mediated Retrograde Transport
Source: PLoS One. 2015 Mar 19;10(3):e0118743. doi: 10.1371/journal.pone.0118743 (PMC4366199; doi:10.1371/journal.pone.0118743)
Supplement: S1 Fig — Schematic of the workflow used to identify Arl1-interacting proteins. The proteomics-based approaches that were used included differential affinity chromatography with immobilized GST-tagged Arl1 (Arl1QL, Arl1 TN and GST control), one-dimensional SDS-PAGE combined with nano-LC-MS/MS, database searches, validation of MS/MS-based peptides and protein identification using Scaffold proteome software. Arl1QL was used as a putative active form of Arl1, whereas Arl1TN was used as a dominant-negative form (inactive form) of Arl1. The plasmid constructions for the GST fusion proteins are described in the S1 document. (PDF) [file pone.0118743.s004.pdf]

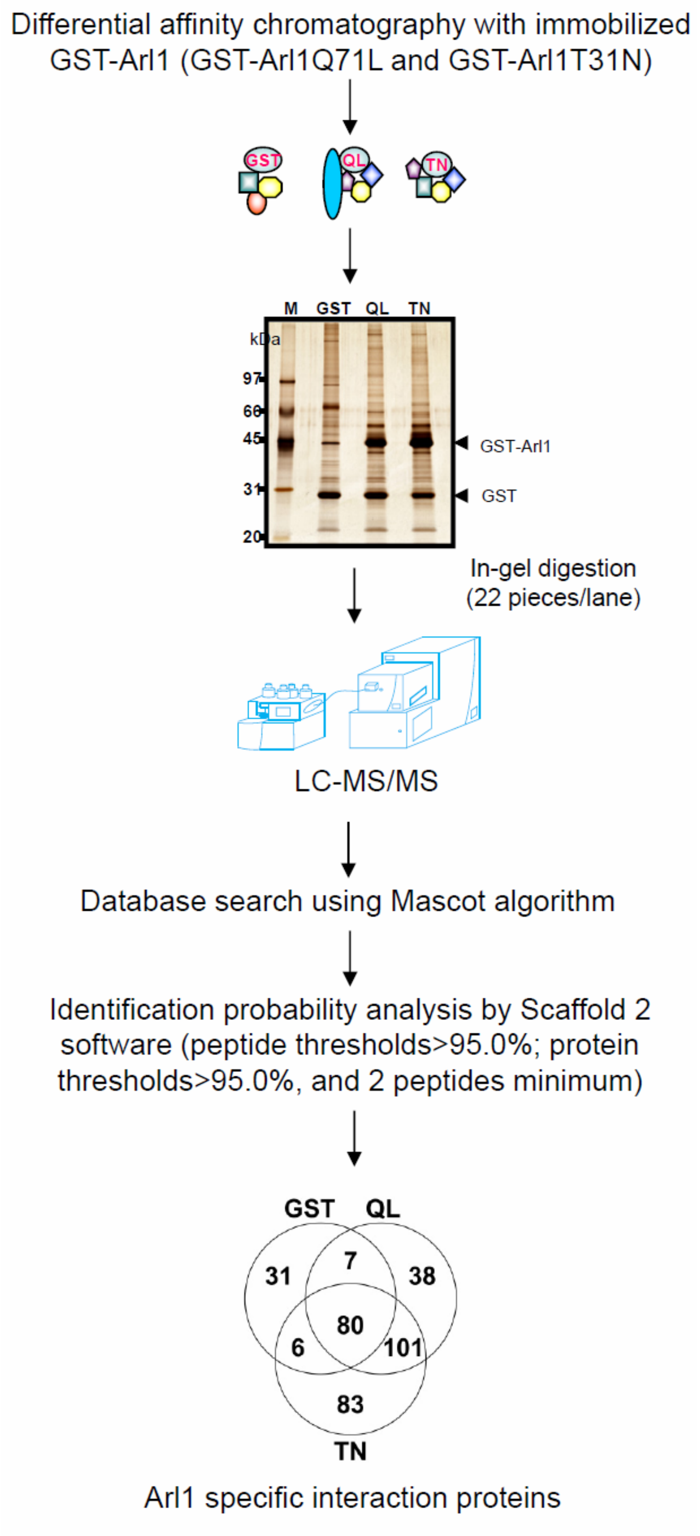
Differential affinity chromatography with immobilized GST-Arl1 (GST-Arl1Q71L and GST-Arl1T31N)
GST
QL
TN
M
GST
QL
TN
kDa
97
66
45
31
20
GST-Arl1
GST
In-gel digestion (22 pieces/lane)
LC-MS/MS
Database search using Mascot algorithm
Identification probability analysis by Scaffold 2 software (peptide thresholds>95.0%; protein thresholds>95.0%, and 2 peptides minimum)
GST
QL
31
7
38
80
6
101
83
TN
Arl1 specific interaction proteins

S1 Fig.
