# Supplementary figures and images for: Arfaptin-1 Negatively Regulates Arl1-Mediated Retrograde Transport
Source: PLoS One. 2015 Mar 19;10(3):e0118743. doi: 10.1371/journal.pone.0118743 (PMC4366199; doi:10.1371/journal.pone.0118743)

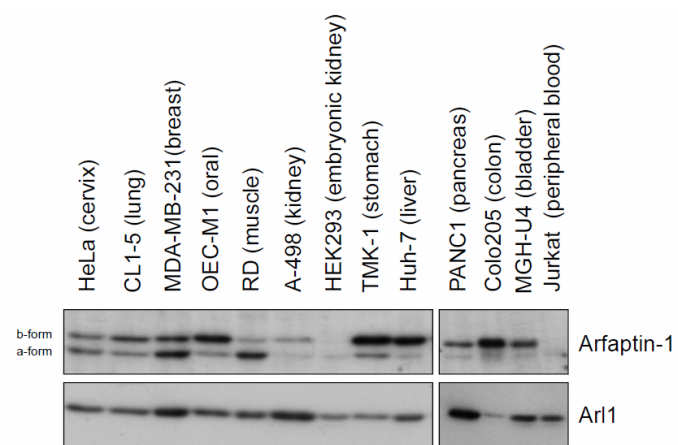

S2 Fig.

Supplement: S2 Fig — Total protein extracts prepared from cancer cell lines (30 μg per lane) were analyzed by western blotting using anti-arfaptin-1 antibody. Actin was used as an internal control (PDF) [file pone.0118743.s005.pdf]

**A**

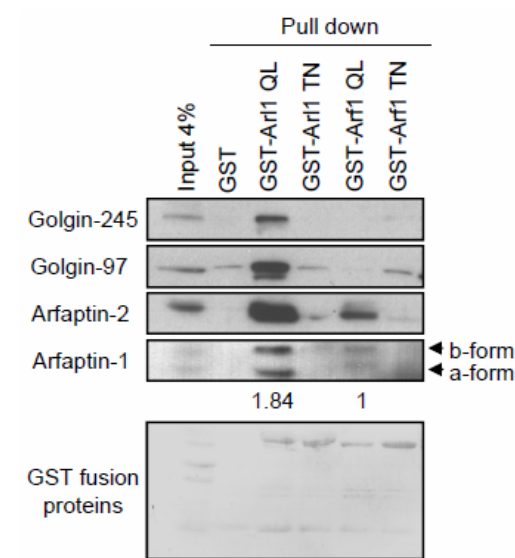

**B**

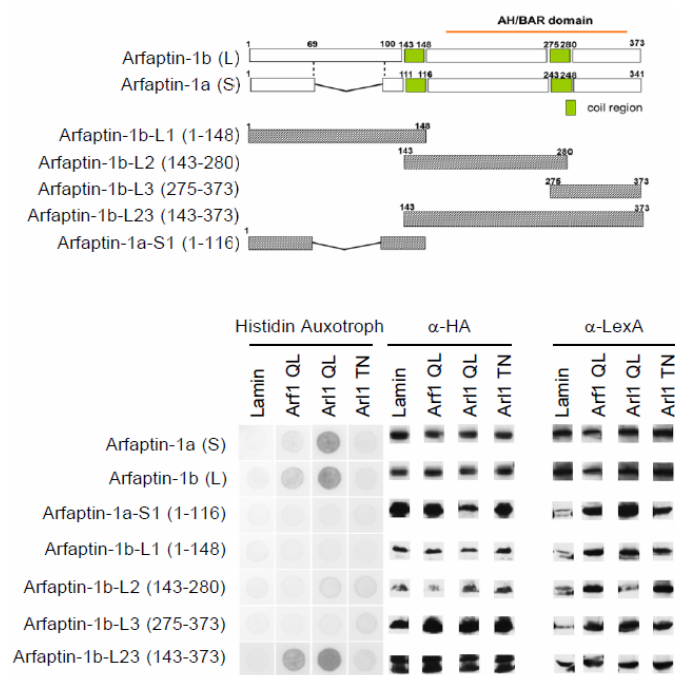

S3 Fig.

Supplement: S3 Fig — (A) Differential affinity chromatography followed by western blotting showed that endogenous golgin-245, golgin-97 and arfaptin-2 and two isoforms of arfaptin-1 interact specifically with Arl1QL. Recombinant GST-Arl1QL, GST-Arl1TN, GST-Arf1QL, GST-Arf1TN and GST were produced by BL21 cells and immobilized on glutathione-sepharose resin. The immobilized GST fusion proteins were incubated with GTPγS and GDP for the QL and TN proteins, respectively. HeLa lysates (2.5 mg) were then incubated with 5 μg of the GST fusion proteins, and the bound proteins were resolved on a 12.5% SDS-PAGE and analyzed by western blotting. The membrane was stained with Coomassie Brilliant Blue to demonstrate equal loading (lower panel), and image quantification revealed that the level of arfaptin-1 bound to Arl1QL was higher (1.84-fold change) than that to Arf1QL. (B) The C-terminal domain of arfaptin-1 (1a and 1b) interacts directly with Arl1QL in a yeast two-hybrid system. Top, diagram of arfaptin-1 and the deletion constructs. Bottom, the small GTPase constructs (Arf1QL, Arl1QL or Arl1TN) fused to the LexA DNA-binding domain and the indicated arfaptin-1 constructs (arfaptin-1a, arfaptin-1b, arfaptin-1a-S1, arfaptin-1b-L1, arfaptin-1b-L2, arfaptin-1b-L3 or arfaptin-1b-L23) fused to the GAL4-activation domain were co-transformed into yeast strain L40. The resulting transformants were plated, and the colonies were screened for histidine auxotrophy. Lamin was used as a negative control. (PDF) [file pone.0118743.s006.pdf]

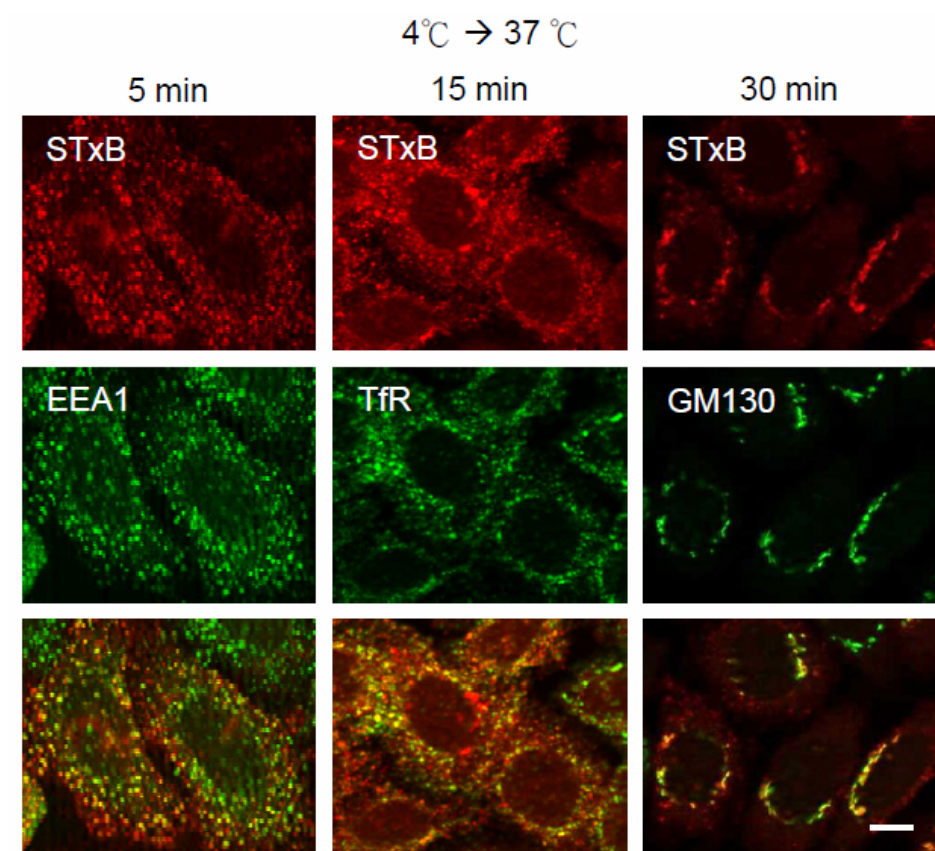

S4 Fig.

Supplement: S4 Fig — HeLa cells were grown on coverslips in a 12-well culture plate for 24 h. Cy3-conjugated STxB was then added to the cultured cells and allowed to bind to the plasma membrane at 4°C for 20 min. The cells were then shifted to 37°C for 5 min, 15 min or 30 min and then fixed and stained with anti-EEA1, anti-TfR and anti-GM130 antibodies. The images were acquired using a Zeiss Apotome fluorescence microscope and Axio vision Rel 4.8 software (Carl Zeiss, Gottingen, Germany). Scale bars, 10 μm. (PDF) [file pone.0118743.s007.pdf]

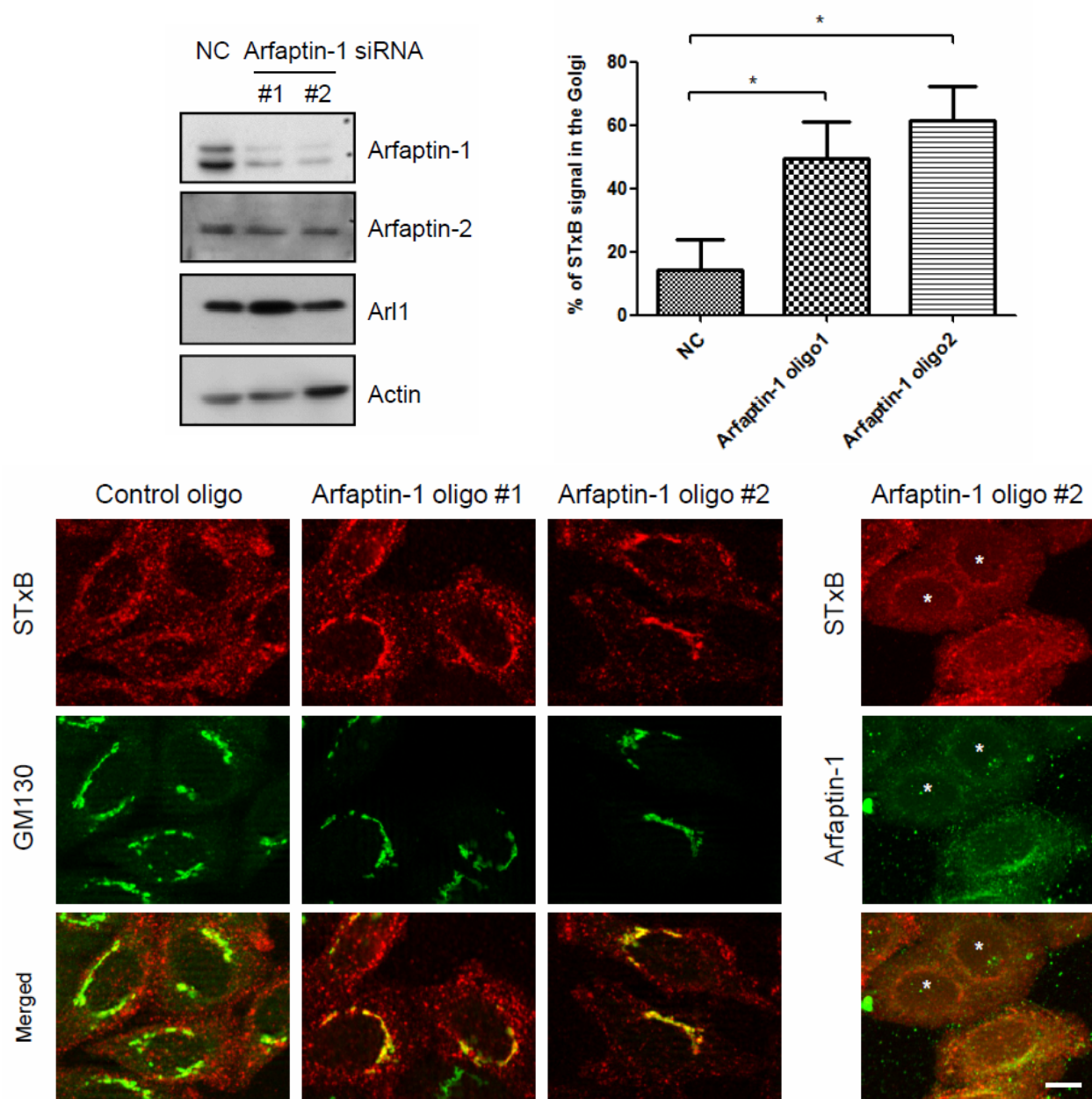

S5 Fig.

Supplement: S5 Fig — HeLa cells were transfected with two unique arfaptin-1 siRNA sequences (oligo #1 or oligo #2) or a control siRNA. After 48 h, the cells were incubated with Cy3-conjugated STxB at 4°C for 20 min and then shifted to 37°C for 15 min. The cells were then fixed and stained with an anti-GM130 antibody. The images were acquired using a Zeiss Apotome fluorescence microscope and Axio vision Rel 4.8 software (Carl Zeiss, Gottingen, Germany). The intensity and area of the STxB (red) and GM130 (green) signals were quantified, and the percentage of the STxB signal in the Golgi was calculated using the following formula: % of STxB signal in the Golgi = total intensity of co-localization of STxB and GM130/total intensity of STxB. The results are presented as the means±SDs; p<0.05 indicates significance, as determined by one-way ANOVA. Scale bars, 10 μm. Asterisks indicate arfaptin-1-knockdown cells. (PDF) [file pone.0118743.s008.pdf]

**A**

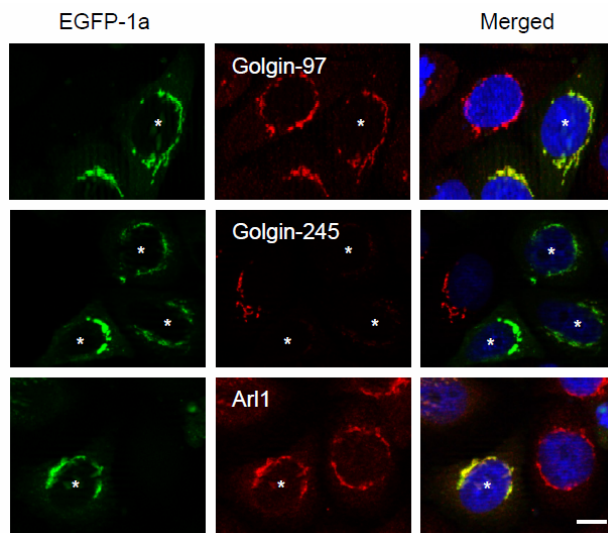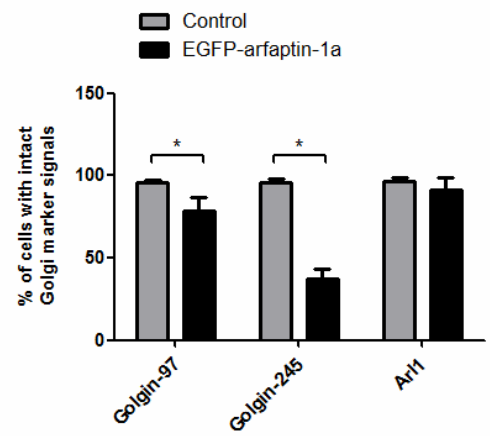

**B**

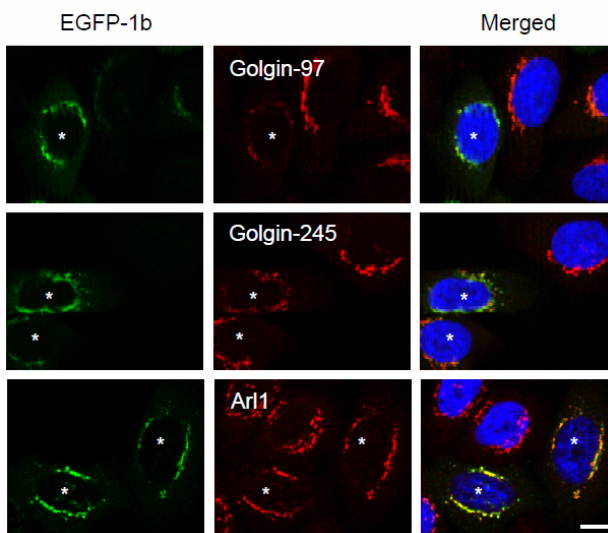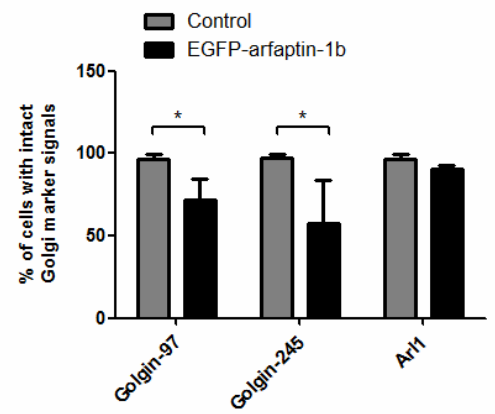

S6 Fig.

Supplement: S6 Fig — HeLa cells were transfected with EGFP-arfaptin-1a (A) or EGFP-arfaptin-1b (B) for 48 h and then stained with anti-golgin-97, anti-golgin-245 and anti-Arl1 antibodies. EGFP-arfaptin-1a and EGFP-arfaptin-1b-expressing cells (n>100 for each experiment) with intact Golgi marker signals were quantified. The results are presented as the means±SDs; p<0.05 indicates significance, as determined by an unpaired Student’s t test. Scale bars, 10 μm. Asterisks indicate EGFP-arfaptin-1a- (A) or EGFP-arfaptin-1b-expressing (B) cells. (PDF) [file pone.0118743.s009.pdf]

**A**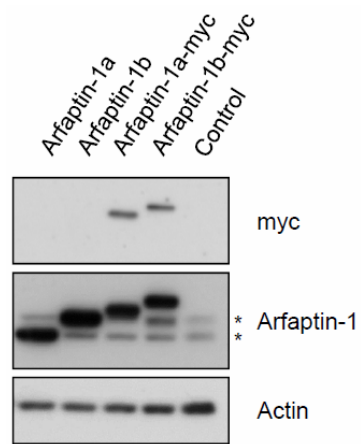**B**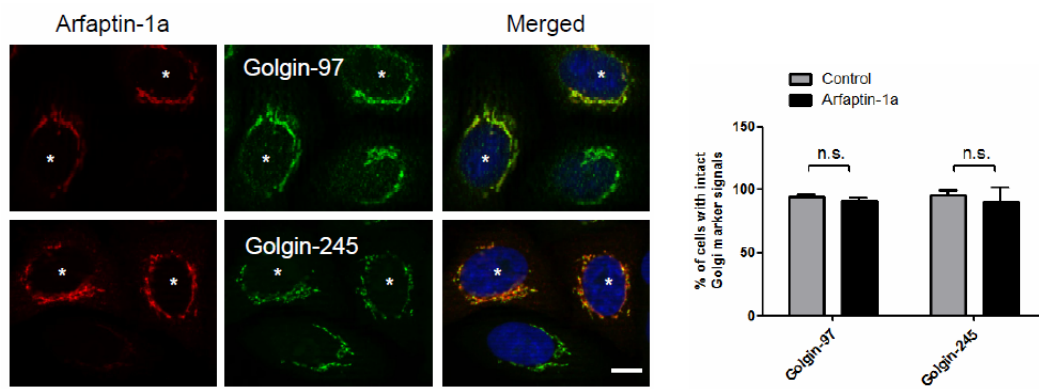**C**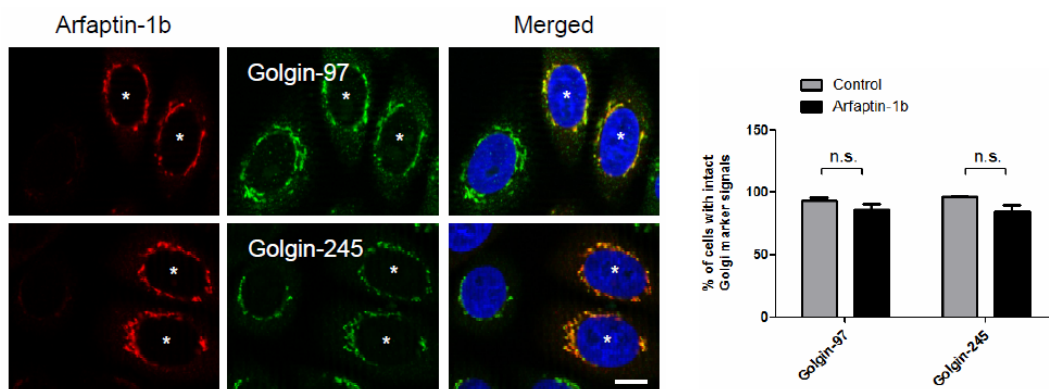**S7 Fig.**

Supplement: S7 Fig — (A) HeLa cells were transfected with tag-free arfaptin-1a, tag-free arfaptin-1b, arfaptin-1a-myc or arfaptin-1b-myc for 48 h, and the cell extracts were analyzed by western blotting using anti-arfaptin-1 and anti-myc as indicated. Actin was use as the internal control. Asterisks indicate endogenous arfaptin-1. (B and C) HeLa cells were transfected with tag-free arfaptin-1a or arfaptin-1b for 48 h and processed for immunofluorescence staining with anti-arfaptin-1, anti-golgin-97 and anti-golgin-245 antibodies as indicated. Arfaptin-1a and arfaptin-1b-expressing cells (n>50 for each experiment) with intact Golgi marker signals were quantified. The results are presented as the means±SDs; p>0.05 indicates non-significance, as determined by an unpaired Student’s t test. Scale bars, 10 μm. Asterisks indicate the tag-free arfaptin-1a- (B) or arfaptin-1b-expressing (C) cells. (PDF) [file pone.0118743.s010.pdf]

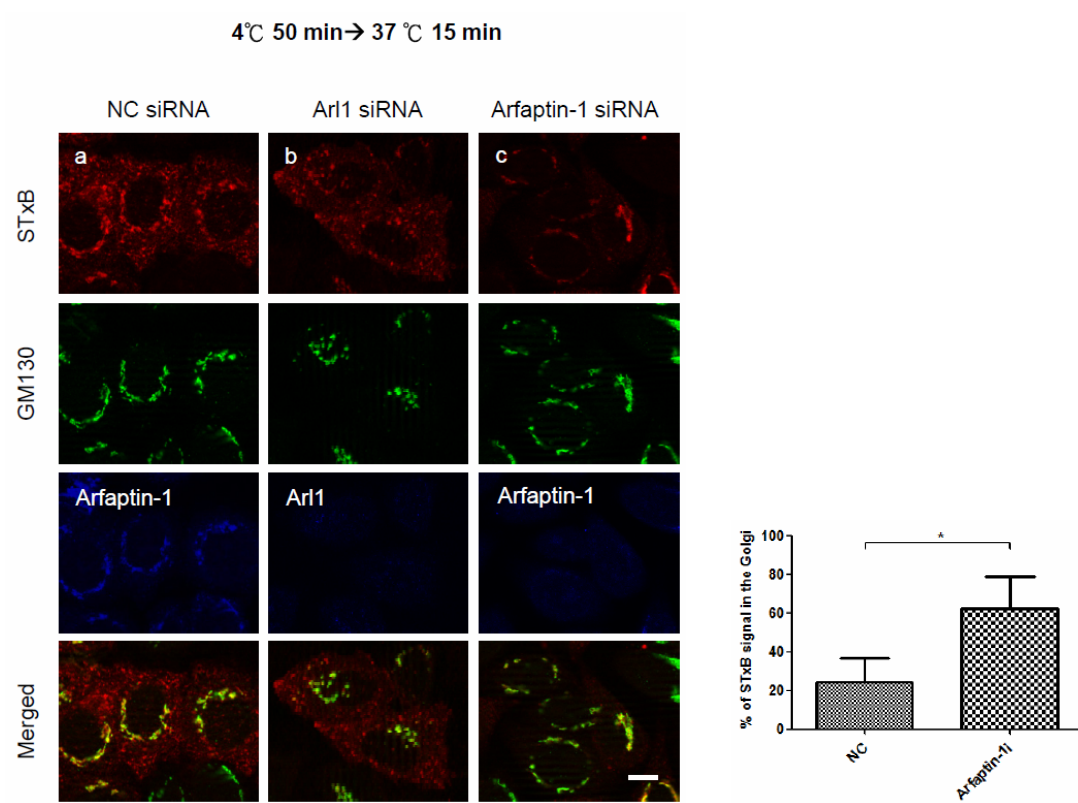

S8 Fig.

Supplement: S8 Fig — HeLa cells were transfected with control siRNA or siRNA specific for Arl1 or arfaptin-1, as indicated. After 48 h, the cells were incubated with Cy3-conjugated STxB at 4°C for 50 min and then shifted to 37°C for 15 min followed by immunofluorescence staining with anti-Arl1, anti-arfaptin-1, or anti-GM130 antibodies as indicated. The intensity and area of the STxB (red) and GM130 (green) signals were quantified (n>50), and the percentage of STxB signal in the Golgi was calculated using the following formula: % of STxB signal in the Golgi = total intensity of co-localization of STxB and GM130/total intensity of STxB. The results are presented as the means±SDs; p<0.05 indicates significance, as assessed by the unpaired Student’s t test. (PDF) [file pone.0118743.s011.pdf]
