# Supplementary material for: Arfaptin-1 Negatively Regulates Arl1-Mediated Retrograde Transport
Source: PLoS One. 2015 Mar 19;10(3):e0118743. doi: 10.1371/journal.pone.0118743 (PMC4366199; doi:10.1371/journal.pone.0118743)
Supplement: S1 Document — (DOC) [file pone.0118743.s012.doc]

**S1 Document. Materials and Methods for Supporting Information**

**Cell culture**

HeLa, MDA-MB-231, RD, Huh-7 and PANC-1 cells were cultured in Dulbecco’s Modified Eagle’s Medium (Gibco, Invitrogen, Carlsbad, CA, USA) supplemented with 10% fetal bovine serum (FBS) plus 100 units/ml penicillin and streptomycin. OEC-M1, CL1-5, TMK-1, Colo205, MGH-U4 and Jurkat cells were maintained in RPMI 1640 medium (Invitrogen) supplemented with 10% FBS plus 100 units/ml penicillin and streptomycin. A-498 and HEK293 cells were cultured in Minimum Essential Media (Invitrogen) supplemented with 10% FBS plus 100 units/ml penicillin and streptomycin. All cells were incubated at 37°C in a humidified 95% air/5% CO_2_ atmosphere.

**GST pull-down assay**

Recombinant GST-fused Arl1QL, Arl1TN, Arf1QL and Arf1TN proteins were expressed in BL21 cells and immobilized on glutathione-sepharose resin (GE Healthcare). The immobilized GST fusion proteins were exchanged for GTPγS (Roche, Mannheim, Germany) or GDP (Sigma-Aldrich, St. Louis, MO, USA) according to the protocol described in Lu *et al .* Briefly, the GST fusion proteins were equilibrated in NE buffer (20 mM HEPES, pH 7.5, 100 mM NaCl, 10 mM EDTA, 5 mM MgCl_2_, and 1 mM DTT) with 0.1% (w/v) sodium cholate and 10 μM GTPγS. After equilibration, the resin was incubated with NE buffer containing 1 mM GTPγS or GDP, 0.1% sodium cholate and 3 mM L-α-dimyristoylphosphatidylcholine for 1.5 h at RT. The resins were then washed and equilibrated by NS buffer (20 mM HEPES, pH 7.5, 100 mM NaCl, 5 mM MgCl_2_, and 1 mM DTT) with 10 μM GTPγS or GDP. The cell lysates prepared from HeLa cells were suspended in NS buffer and precleared by incubating with immobilized GST resin (1 mg HeLa cell lysates/10 μg GST proteins) at 4°C for 2 h. The resulting unbound fractions were collected and incubated with immobilized GST fusion proteins (2.5 mg HeLa cell lysates/5 μg GST fusion proteins) at 4°C overnight. After washing, the bound proteins were resolved on a 12.5% SDS-PAGE gel and analyzed by western blot assays.

**One-dimensional gel electrophoresis and in-gel protein digestion**

Proteins obtained from the pull-down assays were separated on 12.5% SDS/PAGE and stained with Coomassie Brilliant Blue G-250 (AppliChem GmbH, Darmstadt, Germany). The entire gel lane was cut into 22 pieces and subjected to in-gel tryptic digestion, as described previously . Briefly, the gel pieces were de-stained three times, 25 min each, in 50 mM NH_4_HCO_3_/ACN (3:2, v/v) and then dehydrated in ACN and dried in a SpeedVac. The in-gel proteins were reduced with 10 mM dithiothreitol in 25 mM NH_4_HCO_3_ at 56 ^o^C for 45 min, allowed to stand at room temperature (RT) for 10 min, and then alkylated with 55 mM iodoacetamide for 30 min at RT. After the proteins were digested by sequencing-grade modified porcine trypsin (Promega, Madison, WI, USA) overnight at 37 ^o^C, the peptides were extracted from the gel with ACN, brought to a final concentration of 50%, dried in a SpeedVac, and then stored at -20 ^o^C until further use.

**Reverse-phase LC-MS/MS**

The peptide mixture was analyzed with LC-MS/MS using a C18 column coupled with a LTQ-Orbitrap mass spectrometer. Briefly, each peptide mixture was reconstituted in high-performance LC buffer A (0.1% formic acid; Sigma, St. Louis, MO), loaded and trapped on a trap column (Zorbax 300SB-C18, 0.3 × 5 mm; Agilent Technologies) and separated using a 10-cm, analytic C18 column (75-μm inner diameter; New Objective, Woburn, MA) and an ACN gradient in 0.1% formic acid. The LC setup was coupled to a 2D-linear ion trap LTQ-Orbitrap MS (Thermo Fisher, San Jose, CA, USA).

**Database Searching**

All MS and MS/MS data were analyzed and processed using the Mascot algorithm (version 2.2.03, Matrix Science, London) against the Swiss-Prot database (SwissProt_51.6 database, selected for *Homo sapiens*, 15720 entries). The search parameters were set as follows: 0.5 Da for MS/MS tolerance, 10 ppm for MS tolerance, carbamidomethylation (C) as the fixed modification, oxidation (M) as the variable modification, and 1 for missing cleavage. Validation of the MS/MS-based peptides and protein identification was completed using the Scaffold proteome software (version 3.6.5, Proteome Software Inc., Portland, OR), in which the peptide and protein threshold cut-offs were a minimum of 95.0% with a minimum of two peptides.

**Yeast two-hybrid assays**

The yeast strain L40 provides a readout for protein-protein interactions with the use of the LexA DNA-binding domain and GAL4-activation domain system, as described previously . The pBTM116 vector was used to construct pLexA-Arl1QL, pLexA-Arl1TN and pLexA-Arf1QL, in which LexA is fused to the small GTPase (Arl1 and Arf1). Various segments of arfaptin-1 were cloned into the pACT2 vector (Clontech Laboratories, Inc., Mountain View, CA USA) for expression of HA-tagged arfaptin-1. L40 cells were transformed with pLexA-Arl1 and various ARFIPs-pACT2 plasmids using the lithium acetate method (Clontech). Double transformants were plated on synthetic medium lacking histidine, leucine, tryptophan, uracil, and lysine. The plates were incubated at 30°C for 3 days.

**Plasmid constructs**

The plasmids encoding Arl1 and Arf1 were constructed using PCR. Using a two-step PCR procedure, we replaced the codon for Gln71 with the codon for Leu to generate the constructs encoding Arl1QL and Arf1QL. Similar, we replaced the codon for Thr31 with the codon for Asn to generate the constructs encoding Arl1TN and Arf1TN. The Arl1 and Arf1 fragments were subcloned into the pGEX4T vector (GE Healthcare) to generate the glutathione-S-transferase fusion constructs for expression in *E. coli*. The plasmids encoded arfaptin-1a and arfaptin-1b were constructed using the oligonucleotides 5’-GGA TCC TTA TGG CTC AAG AAT CTC C-3’ and 5’- GGA TCC CTG TTC TTC AAG CCA AGA-3’. The amplified fragments of arfaptin-1 (1a and 1b) were ligated into the pGEM-T Easy vector (Promega, Madison, WI, USA) and then subcloned into pEGFP-C2 (Clontech) via BamH1 restriction enzyme sites to generate the EGFP-arfaptin-1 (1a and 1b) fusion protein. Tag-free arfaptin-1a and arfaptin-1b were constructed using oligonucleotides 5’-GGA TCC GGG ATG GCT CAA GAA TCT -3’ and 5’- GGA TCC TTA CTG TTC TTC AAG CCA -3’. The amplified fragments of arfaptin-1 (1a and 1b) were ligated into the pGEM-T Easy vector and then subcloned into pcDNA3.1B via BamH1 restriction enzyme sites.

**References**

Li, C.C., Chiang, T.C., Wu, T.S., Pacheco-Rodriguez, G., Moss, J., and Lee, F.J. (2007). ARL4D recruits cytohesin-2/ARNO to modulate actin remodeling. Molecular biology of the cell *18*, 4420-4437.

Lu, L., Tai, G., and Hong, W. (2005). Interaction of Arl1 GTPase with the GRIP domain of Golgin-245 as assessed by GST (glutathione-S-transferase) pull-down experiments. Methods in enzymology *404*, 432-441.

Yu, C.J., Wang, C.L., Wang, C.I., Chen, C.D., Dan, Y.M., Wu, C.C., Wu, Y.C., Lee, I.N., Tsai, Y.H., Chang, Y.S.*, et al.* (2011). Comprehensive proteome analysis of malignant pleural effusion for lung cancer biomarker discovery by using multidimensional protein identification technology. Journal of proteome research *10*, 4671-4682.
